# Lack of Evidence for the Direct Activation of Endothelial Cells by Adult Female and Microfilarial Excretory-Secretory Products

**DOI:** 10.1371/journal.pone.0022282

**Published:** 2011-08-02

**Authors:** Tiffany Weinkopff, Patrick Lammie

**Affiliations:** 1 Department of Cell Biology, University of Georgia, Athens, Georgia, United States of America; 2 Centers for Disease Control and Prevention, Atlanta, Georgia, Untied States of America; The George Washington University Medical Center, United States of America

## Abstract

Lymphangiectasia (dilation of the lymphatic vessel (LV)) is pathognomonic for lymphatic filariasis. In both infected humans and animal models of infection, lymphangiectasia is not restricted to the site of the worm nest, but is found along the infected vessel. These observations argue that soluble products secreted by the worm could be mediating this effect by activating the lymphatic endothelial cells (LEC) lining the vessel. We tested the ability of filarial Excretory-Secretory products to activate LECs, but were unable to detect a direct effect of the Excretory-Secretory products on the activation of LEC as assessed by a variety of approaches including cellular proliferation, cell surface molecule expression and cytokine and growth factor production (although other mediators used as positive controls did induce these effects). Collectively, these results do not support the hypothesis that Excretory-Secretory products directly activate LECs.

## Introduction

Lymphatic filariasis is a disabling disease transmitted by mosquitoes that infects more than 120 million people throughout the tropics. The infection is caused by filarial nematodes that reside in the lymphatic vasculature. There is a wide spectrum of host response phenotypes; infected individuals often appear to be asymptomatic whereas individuals with lymphedema/elephantiasis are predominantly antigen-negative [1]–[6]. The factors responsible for the progression of disease from infection to clinical lymphedema remain undefined. Even though infected individuals appear asymptomatic, they exhibit subclinical manifestations including lymphangiectasia or dilated lymphatics [1]–[2], [5]. The parasite is thought to be responsible for alterations in the lymphatic endothelium since removal or killing of the worms reverses the dilation [7]–[10]. Furthermore, lymphangiectasia is seen in SCID mice, arguing that the adaptive immune response is not the only driver of this lymphatic pathology [11]–[12]. Lymphatic dilation is greatest near the site of the worm nest, but it is not restricted to the site of the worm nest and is found along the length of infected vessels suggesting that a soluble product secreted by the worm may be mediating these effects [13]–[14]. These findings support the conclusion that the presence of living adult worms and their ES products alters LV tissue. Taken together, these observations suggest that LECs are the prime targets of parasite-derived factors which initiate the development of clinical pathology [15].

We and others have previously characterized the protein constituents making up the filarial ES products released by the worm [unpublished data], [ 16]–[18], but the biological effects of these molecules have not been fully elucidated. Given the altered pathology along the length of the infected vessel and intimate relationship between the parasite and the endothelial cells lining the LVs, worm ES products may be contributing to the pathogenesis of disease. Therefore, we examined the biological effects of filarial ES products on LECs. LECs were stimulated with filarial ES products and assayed for changes in differentiation, activation and proliferation. Changes in cell surface marker expression profiles, the presence of phosphorylated cell signaling molecules, gene expression and growth factor production were used to characterize the LEC response to worm ES products.

## Materials and Methods

### Parasite materials and collection of ES products

*Brugia malayi* adult worms and microfilariae were collected from the peritoneal cavity of infected jirds, *Meriones unguiculatus*, that were obtained from the NIAID Filariasis Repository at the University of Georgia (Athens, GA). For the collection of ES products, 50 live adult female worms were cultured *in vitro* for 7 days at 37 C in 10 mL serum-free RPMI 1640 media (GIBCO) supplemented with 2 mM L-glutamine and antibiotics (100 U/mL penicillin and 100 µg/mL streptomycin). Supernatants were collected and fresh medium added daily. The microfilariae were resuspended in PBS and counted to ensure worm viability. Supernatants containing the ES products were centrifuged at 1000× g for 10 min to remove the microfilariae and then concentrated with a Centricon filter (Millipore, Bedford, MA) to a volume of ∼300 µL. ES products were stored at 4 C until further use. Prior to cell stimulations with ES products, ES products were filtered using 0.45 µm Millex-HA syringe filters (Millipore, Carrigtwohill, Co. Cork, Ireland) and used in a dose-dependent (diluted 1∶10, 1∶50, 1∶100). All batches of ES products were tested for endotoxin activity using the Limulus Amebocyte Lysate QCL-1000 assay (Lonza, Walkersville, MD) and ES products were only used for cell stimulations when endotoxin concentrations ≤0.1 eu/mL.

For the preparation of adult worm and microfilariae extract, worms were stored in phosphate-buffered saline (PBS) at −80 C until thawed. Crude worms were cut with scissors, resuspended in 3 ml PBS and ultrasonicated (Heat Systems-Ultrasonics, Inc., Plainview, New York; Model W-225 with standard tip) for 30 min at 4°C. The material was centrifuged for 10 min at 15,000 *g* at 4 C and the protein concentration of the supernatant was determined by the bicinchoninic acid protein assay (Pierce, Rockford, Illinois).

### Culture of ECs

Primary adult human dermal lymphatic microvascular endothelial cells (HMVEC-dLy) (cat. CC-2810T25) were purchased from Lonza Clonetics (Walkersville, MD) and maintained in EGM-2MV Bulletkit media (cat. CC-3202) from Lonza Clonetics. Cells were maintained according to manufacturer's instructions and used between passages 4 through 8. In addition, well-characterized cell lines such as the human dermal microvascular endothelial cell line, HMEC-1 [19], a human umbilical vein endothelial cell line (HUVEC) and a bovine endothelial cell line (BOVEC) were obtained from ATCC through the CDC and used in parallel experiments. hTERT-HDLEC cells were kindly provided by Drs. Katy Baty and David Finegold at the University of Pittsburgh. These cells are composed of primary human dermal microvascular endothelial cells (HDMVEC) that were transfected with a retrovirus containing the coding region of human telomerase reverse transcriptase (hTERT) to provide a primary lymphatic endothelial cell line (hTERT-HDLEC) with an extended lifespan [20]. HMEC-1, HUVEC and BOVEC cell lines were obtained from the CDC and maintained in EBM131 media containing endothelial basal media (MCDB131; GIBCO) supplemented with 15% FBS, 10 ng/ml epidermal growth factor, 1 µg/ml hydrocortisone, 90 µg/mL heparin, 2 mM L-glutamine and antibiotics (100 U/mL penicillin and 100 µg/mL streptomycin). For starvation experiments, cells were cultured in MCDB131 media with L-glutamine and antibiotics. hTERT-HDLECs were maintained in the same EBM131 media as HMECs, but EndoGro (Vec Technologies, Rensselaer, NY) was added according to manufacturer's instructions. Cell lines were detached from flasks using 0.02% versene and 0.2% trypsin treatment.

### Cellular proliferation

Cells were seeded into a 96-well plate and allowed to adhere overnight. If cells were starved, cells were cultured in MCDB131 starvation media for 4 or 24 hours prior to stimulation in a number of different media and in the presence or absence of VEGF (1, 10 or 100 ng/mL) or ES products (diluted 1∶10 to 1∶250) for 72 hours. The cell types and culture conditions used to demonstrate EC proliferation are summarized in Table 1. Proliferation was assayed by adding 0.5 µCi [^3^H] thymidine/well and measuring the incorporation of tritiated thymidine over the last 8 or 16 hours of culture using a 1205 Betaplate liquid scintillation counter (Wallac, Perkin-Elmer, Waltham, MA).

**Table 1:** *In vitro* cell culture conditions for EC proliferation experiments.

Cell type	Media used for 72 hour stimulation	Cell No. (range)	Serum (range)	Starvation time in MCDB131 (serum-free)
BOVEC	EBM131 or MCDB131	4×10^3^–2×10^4^	0 or 15% FBS	24 hours
HMEC	EBM131 or MCDB131	4×10^3^–2×10^4^	0 or 15% FBS	No starvation; 4 or 24 hour starvation
hTERT-HDLEC	EBM131 + EndoGro	1×10^4^	15% FBS	No starvation
LEC	MCDB131 or EGM-2MV	2×10^3^–5×10^4^	1.25–10% and 15% FBS1.25–10% NHS	No starvation; 4 or 24 hour starvation

### Detection of NFκB phosphorylation

LECs, HMECs or HUVECs were seeded in a 6-well plate in EGM-2MV or EBM131 media and grown until confluent. Cells were starved for 2.5 hours with MCDB131 media heated to 37 C. Cells were stimulated in the warmed media for 15 min (LECs in EGM-2MV; HUVECs in EGM131) and 30 min (HMEC in EGM131) and lysed with 25 µL/mL 1 M Tris-HCl (pH 7.5), 50 µL/mL 20% SDS, 925 µL/mL H_2_O, 200 µg/mL ethylenediaminetetraacetic acid (EDTA), 1 µg/mL leupeptin, 1 µg/mL pepstatin A, 200 µg/mL Pefabloc SC (Roche, Indianapolis, IN) and Phosphatase Inhibitor Cocktail Set II (1∶100; Calbiochem, La Jolla, CA). Lysate protein concentrations were determined by BCA (Pierce); 2.5 µg (LEC) and 5 or 7.5 µg (HMEC) protein per lane was separated in a 12% SDS-PAGE gel and electrophoretically transferred onto Immobilon-P PVDF membranes (Millipore).

Throughout the western blotting, PBS plus 0.3% Tween was used in each step. Following transfer, the membranes were incubated overnight at 4 C with primary antibodies purchased from Cell Signaling Technology (Danvers, MA) including rabbit anti-phospho-p44/42 MAPK (1∶300; cat. 4376S), rabbit anti-phospho-pAkt (1∶300; cat. 4056), rabbit anti-phospho-pNFκB p105 (1∶300; cat. 4884) or rabbit anti-p44/42 MAPK (1∶300; cat. 9102), rabbit anti-Akt (1∶300; cat. 9272), rabbit anti-NFκB p105/p50 (1∶300; cat. 3035) or mouse anti-β-tubulin (1∶4000; cat. T6074; Sigma). The membranes were washed and incubated for 1 hour with HRP-conjugated goat anti-rabbit IgG antibody (1∶1000; cat. 7074; Cell Signaling Technology) or HRP-conjugated goat anti-mouse IgG antibody (1∶1000; cat. 12–349; Millipore), as appropriate. After a final wash, the bound conjugates were visualized by 3,3′-diaminobenzidine (Sigma) as the chromagenic substrate.

### Cytokine and growth factor production

LECs were plated in a 6-well plate at 3×10^5^ cells per well in 1.5 mL EGM-2MV media and the monolayer was grown until confluent. LECs were then stimulated with or without 100 ng/mL LPS, 10 ng/mL TNFα or ES diluted at 1∶10 for 72 hours. Cell culture supernatants were collected and stored at −80 C until further use. For parallel experiments examining the effects of filarial crude worm and microfilariae extracts, 3×10^5^ LECs were plated in endothelial basal media (EBM, Lonza Clonetics), stimulated with or without *Brugia* ES products (1∶10), *Brugia* adult worm extract (10 µg/mL), *Brugia* microfilarial extract (25 µg/mL or 10 µg/mL) for 24 hrs and supernatants were collected. Supernatants were analyzed for IL-2, IL-4, IL-5, IL-6, IL-8, IL-10, IL-12p70, IL-13, IFNγ, TNFα and VEGF-A by luminex technology using the Bio-Plex Pro multiplex suspension array system (Bio-Rad, Hercules, CA) according to the manufacturer's instructions. Data were analyzed by the Bio-Plex Manager software version 4.1.1 and concentrations were calculated based on a standard curve derived from recombinant cytokine standards. If the cytokine level in the sample was lower than the lowest value on the standard curve, which occurred in multiple stimulations, the lowest value of the standard curve was reported for that data point.

For the production of VEGF-C and VEGF-D, LECs were plated in a 24-well plate at 2.5×10^4^ cells per well in 500 µL EGM-2MV media and allowed to adhere for 24 hours at which point the monolayer was also confluent. LECs were starved for 24 hours in MCDB131 media and then stimulated in 1.5 mL EGM-2MV media with or without 10 ng/mL TNFα or ES diluted at 1∶10 for 72 hrs. Cell culture supernatants were collected and stored at −80 C until further use. VEGF-C and VEGF-D production were analyzed by the Quantikine Immunoassay kits (R&D, Minneapolis, MN) as directed by the manufacturer. All cytokine and growth factor production experiments were carried out in triplicate.

### Flow cytometry

LECs, HMECs or HUVECs were grown to confluence in their appropriate media and either starved for 24 hours in MCDB131 or directly stimulated in the appropriate media with or without 100 ng/mL LPS, 10 ng/mL TNFα or ES (diluted at 1∶10 and 1∶50) for 24 hours. Cells were removed from the flask by trypsinization and stained for 30 min on ice in the dark according to manufacturers' directions using unconjugated rabbit anti-human VEGFR-3 (cat. 102-PA18AG; ReliaTech, Braunschweig, Germany), mouse anti-human podoplanin (cat. 101-M40; ReliaTech) and rabbit anti-human LYVE-1 (cat. 102-PA50S; ReliaTech) primary antibodies and followed by secondary staining using FITC-labeled goat anti-rabbit (cat. 554020; BD Pharmingen) and PE-labeled goat anti-mouse antibodies (cat. 550589; BD Pharmingen). Cells were also stained using primary antibodies purchased from BD Pharmingen including conjugated PE-Cy5-labeled anti-human ICAM-1 (cat. 555512), FITC-labeled anti-human VCAM-1 (cat. 551146) and PE-Cy5-labeled anti-human E-selectin (cat. 550040). Cell events were acquired and analyzed on a BD FACScan flow cytometer (BD Biosciences, San Jose, CA).

## Results

**Figure 1:**
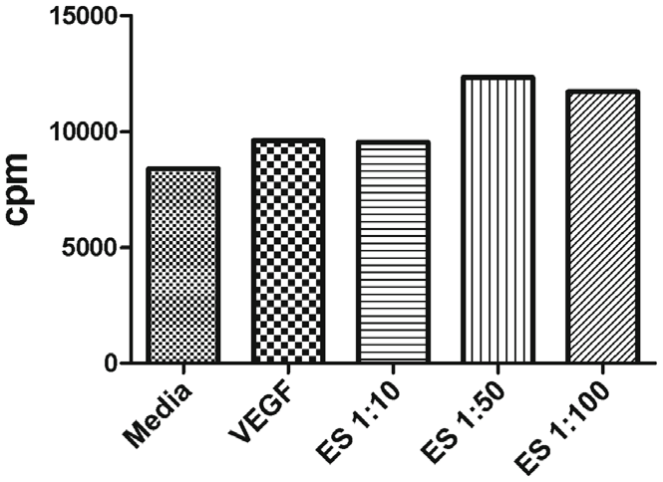
Proliferation of LECs in response to filarial ES products. 5×10^4^ LECs were plated in a 96 well plate in EBM-2MV and allowed to adhere for 24 hours. Cells were starved for 24 hours in MCBD media and then stimulated with or without VEGF (10 ng/mL) or a dilution series of filarial ES products from 1∶10 to 1∶100 in MCDB media containing 5% NHS for 72 hours. Thymidine incorporation was measured. Data seen here is one representative LEC proliferation experiment. Various cell lines, cell numbers, starvation conditions, media types and serum types were utilized for optimization of this assay but we never detected reproducible proliferation rates in response to ES products.

To model the intimate interaction between worm ES products and the LECs in filarial infection, an *in vitro* model system was established in which LECs were exposed to filarial ES products released by the parasite. Patients with active infection exhibit lymphangiectasia whereby LVs are dilated, but the mechanism causing this alteration is not known. The diameter of an infected LV is increased and this dilation could be a result of an increase in the number of LECs lining the lymphatic lumen, so we measured the proliferation of LECs stimulated with filarial ES products. To test this, decreasing concentrations (1∶10 to 1∶100) of ES products were added to LECs as well as the hTERT-HDLEC and HMEC-1 cell lines *in vitro* for various time periods and proliferation was compared to cells cultured in media alone. Under various starvation conditions and in the presence of a range of natural human or fetal bovine serum concentrations (1.25–10%) in multiple EC media (including EGM-2MV, EBM131 and basal MCDB131), we were not able to detect reproducible LEC proliferation in response to worm ES products; a representative experiment using LECs is shown in Figure 1.

When we did not detect increased rates of EC proliferation in response to worm ES products, we examined the ECs for earlier activation events such as the phosphorylation of cell signaling molecules. ECs including LECs, HMECs and HUVECs were stimulated with or without ES (1∶10) or TNFα and the phosphorylation of NFκB, Akt and MAPK ERK1/2 was compared to cells cultured in media alone at the same time points. In pilot experiments, we defined optimal time points for detection of the phosphorylated molecules in response to TNFα (data not shown). We were not able to detect reproducible phosphorylation of either NFκB or Akt in response to worm ES products. We did detect the phosphorylation of the MAPK ERK1/2 p44/42 subunit in response to worm ES products, but this was a transient response that was not associated with any downstream events in our analyses.

**Figure 2:**
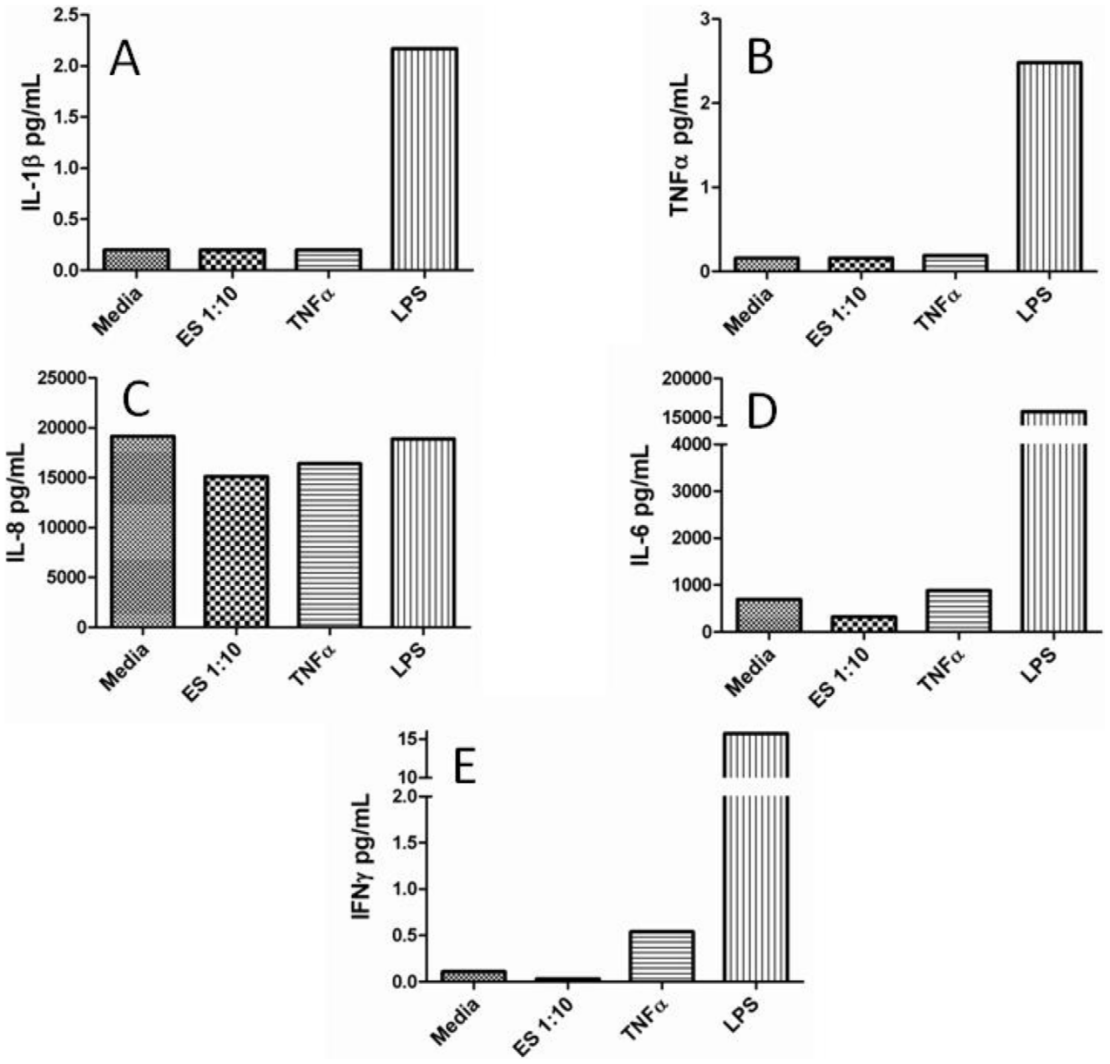
Cytokine production by LECs in response to filarial ES products. 3×10^5^ LECs were plated in EGM-2MV media and allowed to come to confluence. Cells were then stimulated in EGM-2MV media with or without ES (1∶10), LPS (100 ng/mL) or TNFα (10 ng/mL) for 72 hrs and supernatants were harvested and assessed for cytokine production including IL-1β (A), TNFα (B), IL-8 (C), IL-6 (D) and IFNγ (E) by luminex bead technology. Experiments were completed in triplicate and this figure includes representative data from one experiment.

**Figure 3:**
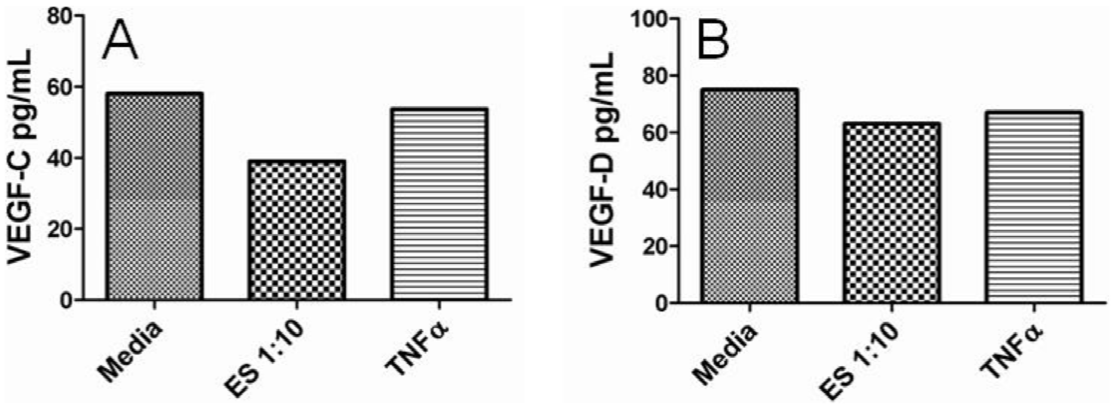
Growth factor production by LECs in response to filarial ES products. 2×10^4^ LECs were plated in EGM-2MV media and allowed to adhere for 24 hours. Cells were then starved for 24 hours in MCDB-131 media prior to stimulation for 24 hours in EGM-2MV media with or without ES (1∶10) or TNFα (10 ng/mL). Supernatants were harvested and assessed for growth factor production including VEGF-C (A) and VEGF-D (B) by ELISA. Experiments were completed in triplicate and this figure includes representative data from one experiment.

Cytokine and growth factor production by LECs in response to worm ES products as well as LPS and/or TNFα were also assessed to identify any downstream cellular activation events that might be associated with the phosphorylation of cell signaling molecules. After 72 hours of culture, IL-1β, IL-6, IFNγ and TNFα production were only detected in supernatants from LECs stimulated with LPS but not ES or TNFα; IL-8 production was similar for all experimental parameters (Fig. 2). IL-2, IL-12, and IL-13 were not induced by ES, LPS or TNFα and IL-4, IL-5 and IL-10 production were below the detection limits of the luminex assay regardless of stimulus (data not shown). It should be noted that LECs stimulated with microfilariae crude extract significantly stimulated the production of GM-CSF and IL-2, suggesting that LECs can respond to filarial-specific products extracted from dead parasites (Fig. S1). For growth factor production, similar levels of VEGF-C and VEGF-D were detected in both stimulated and unstimulated LEC supernatants after 72 hours (Fig. 3) and VEGF production was below the limits of detection by the luminex assay (data not shown).

**Figure 4:**
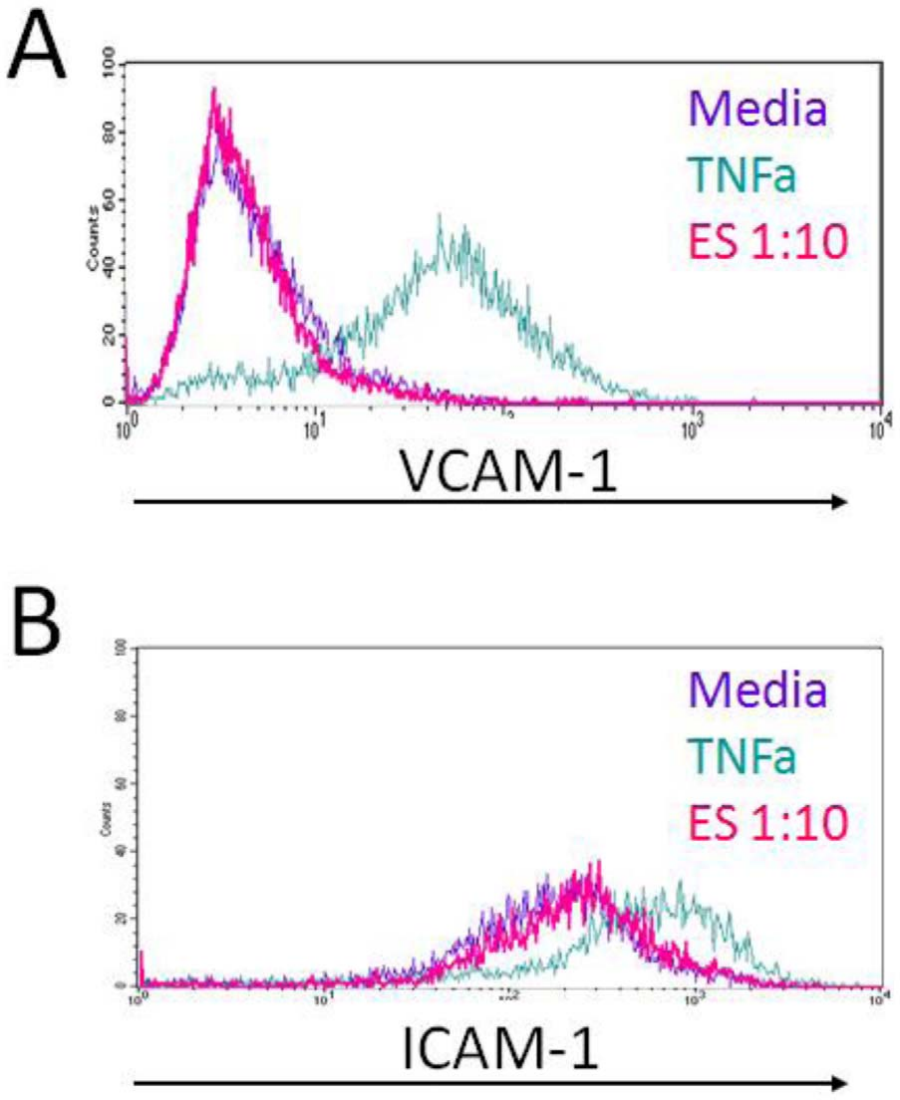
Surface expression of adhesion molecules in response to worm ES products. LECs were grown to confluence in EGM-2MV media then cells were stimulated for 24 hours in EGM-2MV media with or without ES (1∶10) or TNFα (10 ng/mL). LECs were harvested and subjected to flow cytometry analysis for the expression of ICAM-1 or VCAM-1. This figure includes representative data from one experiment.

Endothelial cells were also examined for changes in the expression of cell surface molecules in response to filarial ES products. ICAM-1 and VCAM-1 are adhesion molecules known to be expressed on the surface of blood vascular endothelial cells and up-regulated in response to inflammatory stimuli; therefore, we measured the expression of ICAM-1 and VCAM-1 on LECs as well as HMECs and HUVECs that were stimulated with worm ES products for 24 hours. We did not detect an up-regulation of ICAM-1 and VCAM-1expression in any of the ECs tested in response to worm ES but we did detect an up-regulation of these adhesion molecules in response to TNFα (Fig. 4).

We also analyzed LECs for changes in LEC-specific surface marker expression by flow cytometry. We did not detect and up-regulation of LYVE-1, podoplanin or VEGFR-3 surface expression on LECs that were stimulated with worm ES products or TNFα compared with the LECs cultured in media alone (data not shown).

## Discussion

The association of lymphangiectasia with the presence of active filarial infection argues that, soluble parasite factors may be mediating the effect *in vivo*. We originally hypothesized that the ES products of the worms were activating the lymphatic endothelium; however, we were not able to detect a direct effect of the ES products on the activation of LECs as summarized in Table 2. We considered that the lymphangiectasia could be due to an increase in the rate of LEC proliferation, but we did not see increased proliferation of these cells in repeated assays. In addition, the LECs did not appear to be stimulated by ES products as assessed by expression of cell surface molecules or production of growth factors or cytokines. The positive controls in these assays induced the expected responses, so the cells were viable and functional; however, the filarial ES products did not induce detectable responses by LECs.

**Table 2:** Incubation of LECs with *Brugia* ES fails to induce cellular activation as measured by a variety of parameters.

Activation Event	Measurement	Cell Type
Proliferation	Thymidine incorporation	HMEC, hTERT-hdLEC^c^, BOVEC or LEC
Cell signaling	Western blot for: MAPK ERK1/2, Akt, NFκB	HMEC, HUVEC, LEC^d^
Cell surface molecule expression^a^^,^ b	Flow cytometry for: ICAM-1, VCAM-1, E-selectin, VEGFR-3, LYVE-1, podoplanin or Prox-1	HMEC, HUVEC (VCAM-1 only) or LEC
Growth factor production^a^^,^ b	ELISA for: VEGF (VEGF-A), VEGF-C or VEGF-D	HMEC or LEC
Cytokine production^a^^,^ b	Bioplex for: IL-1β, IL-2, IL-4, IL-5, IL-6, IL-8, IL-10, IL-12p, IL-13, IFNγ, TNFα	LEC

a LPS (100 ng/ml) used as a positive control;

b TNFα (10 ng/mL) used as a positive control;

c hTERT-hdLEC [20];

d Phosphorylation of MAPK observed in LECs but not other cell type.

Some authors have proposed that lymphatic filarial parasites induce endothelial and connective tissue proliferation *in vivo* which in turn causes the thickening of the endothelium [21]–[22]. The cuboidal ECs seen in *B. malayi*-infected nude mice suggest that a multiplication of LECs may also contribute to lymphangiectasia [23]. However, this phenomenon could not be reproduced *in vitro* with human umbilical vein endothelial cells [15]. We repeated these *in vitro* experiments with LECs, but we were still not able to detect an increase in EC proliferation in response to worm ES products under the culture conditions that we employed. In these experiments, we used ES products from adult female worms because of their greater abundance compared to ES products of male worms. We should also note that the ES products used to stimulate LECs do contain ES products of microfilariae, as this parasite stage is also released by the female worm *in vitro* at the same time that the female worm secretes ES products. We did not attempt to separate the adult female and microfilarial ES products as both may be found in the infected lymphatic vessels potentially inducing lymphatic dilation. We reasoned that ES products from both stages would be best to reproduce the biological environment *in vivo*. In preliminary experiments, we also attempted to co-culture living adult worms with LEC monolayers, but the active movement of the living adult worms disrupted the LEC monolayer, preventing our efforts to address the direct effect of the living parasites on LEC function.

In our hands, we were not able to demonstrate reproducible LEC proliferation in response to the positive control VEGF. The lack of a robust *in vitro* proliferation response to VEGF is not uncommon and many responses only exhibit <50% increase over ECs stimulated in media alone [24]–[26]. VEGF did not induce a significant increase in the proliferation of LECs even at lower seeding numbers, other serum concentrations or other stimulation times suggesting VEGF may not be the best positive control for *in vitro* proliferation studies using LECs, but may be better suited for vascular ECs. The lack of proliferation seen in the HMVEC-dLy cells upon stimulation with VEGF may result from the commercial optimization of culture conditions for these cells with VEGF. According to the manufacturer's instructions, these primary LECs require VEGF for routine culture so the ability of VEGF to induce LEC proliferation may be diminished.

In general and in our cultures, the lack of LEC proliferation is most likely related to the stringency of the culture conditions required for cell growth. However, Bennuru and Nutman demonstrated microfilariae-induced LEC differentiation as measured by tubule formation, suggesting that further investigation is needed to address the potential role of microfilariae in altering lymphatic pathology [27]. In parallel experiments, microfilariae crude worm extract stimulated the production of various lymphangiogenic and immunologic mediators such as GM-CSF and IL-2 by LECs confirming the ability of LECs to respond to filarial-specific products but these mediators were not produced at elevated levels by LECs exposed to filarial ES products (Fig. S1). This suggests that LECs exhibit differential responses to individual parasite stages or products. Furthermore, given that ES products contain products from both adult worms as well as microfilariae, and there was not an increase in cytokine production by LECs in response to ES products, these data also demonstrate a differential response between microfilarial ES products and microfilarial crude worm extract. Taken together, these data suggest that EC proliferation under these culture conditions does not result from direct exposure to adult female worm and microfilariae ES products; however, EC proliferation may require unidentified culture conditions, other parasite stages or involve other factors, such as accessory host cells or host-derived products.

It should be noted that subsequent experiments in our lab revealed that filarial ES products stimulated human peripheral blood mononuclear cells (PBMCs), and specifically monocytes, to produce lymphangiogenic mediators arguing that the lack of measurable effects on LECs in response to worm ES products is not a result of protein concentration (unpublished data, Weinkopff et. al). Lymphangiogenic mediators produced by PBMCs in response to stimulation with filarial ES products were able to alter the behavior of LECs *in vitro* and *in vivo* as measured by tubule formation suggesting that LECs are indirectly activated by filarial ES products through the production of lymphangiogenic mediators from PBMCs (unpublished data, Weinkopff et. al). The involvement of host-derived products mediating lymphangiectasia is supported by the observation that serum from infected individuals can induce LEC proliferation [27].

Even though we did not identify a reproducible activation event induced by worm ES, we did demonstrate robust responses of LECs to the positive controls, TNFα and LPS, in multiple assays. Here, we report that both TNFα and LPS stimulate LECs to activate cell signaling events, up-regulate cell surface adhesion molecules and induce growth factor and cytokine production. TNFα (10 ng/mL) has been shown to increase ICAM-1 and VCAM-1 expression on LECs by flow cytometry [28] and TNFα has also been reported to enhance ICAM-1 and VCAM-1 transcript levels [29]. In our hands LPS (100 ng/mL) induced the production of IL-1β, IL-6 and TNFα by LECs compared to unstimulated LECs (Fig. 2) and similar results were reported by Pegu et al. [29]. Collectively, these data argue that even though we did not detect an ES-induced activation event in LECs, our cells were viable and we had established the appropriate positive controls to induce and detect optimum responses.

In conclusion, we were not able to demonstrate a direct activation of LECs by filarial ES products. The lack of evidence for a direct activation event may be explained by the limitations of an *in vitro* culture model system. Given the longevity of filarial infections, worms can exist in LVs for years where they release soluble factors that may gradually alter the lymphatic endothelium. In our *in vitro* cultures, we only carried out the LEC-stimulations with ES products for 72 hours; this may not be enough time to recreate the effects seen in infected vessels. The negative results may also suggest that a more complicated network is established between the parasite and the host and the LECs may be indirectly activated through a host accessory cell or its mediators.

## Supporting Information

Figure S1 **Evidence for the production of immunologic mediators by LECs.** 3×10^5^ LECs were plated in EBM and stimulated with or without *Brugia* ES products (1∶10), *Brugia* adult worm extract (10 µg/mL), *Brugia* microfilarial extract (25 µg/mL or 10 µg/mL) for 24 hrs and supernatants were harvested and assessed for cytokine production including GM-CSF and IL-2 by luminex bead technology. Experiments were completed in triplicate and this figure includes data from one experiment.
(TIFF)
