# Supplementary figures and images for: Lack of Evidence for the Direct Activation of Endothelial Cells by Adult Female and Microfilarial Excretory-Secretory Products
Source: PLoS One. 2011 Aug 2;6(8):e22282. doi: 10.1371/journal.pone.0022282 (PMC3149047; doi:10.1371/journal.pone.0022282)

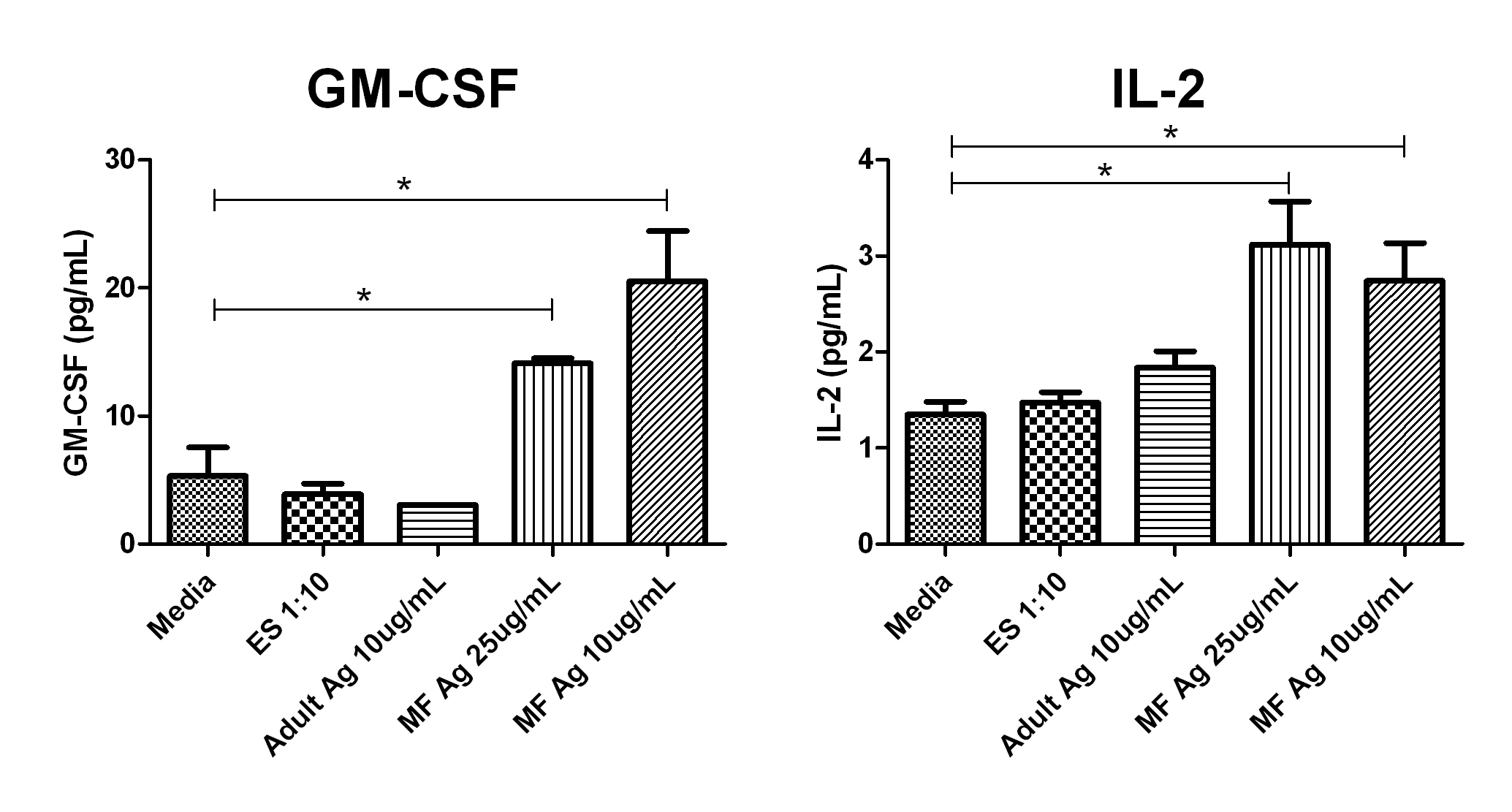

Supplement: Figure S1 — Evidence for the production of immunologic mediators by LECs. 3×10^5^ LECs were plated in EBM and stimulated with or without Brugia ES products (1∶10), Brugia adult worm extract (10 µg/mL), Brugia microfilarial extract (25 µg/mL or 10 µg/mL) for 24 hrs and supernatants were harvested and assessed for cytokine production including GM-CSF and IL-2 by luminex bead technology. Experiments were completed in triplicate and this figure includes data from one experiment. (TIFF) [file pone.0022282.s001.tiff]
